# New methods of posturographic data analysis may improve the diagnostic value of static posturography in multiple sclerosis

**DOI:** 10.1016/j.heliyon.2021.e06190

**Published:** 2021-02-11

**Authors:** Janusz W. Błaszczyk, Joanna Cieślińska-Świder, Renata Orawiec

**Affiliations:** a Department of Human Motor Behavior, The Jerzy Kukuczka Academy of Physical Education, Katowice, Poland; b Department of Physiotherapy of the Nervous System and the Musculoskeletal System, The Jerzy Kukuczka Academy of Physical Education, Katowice, Poland; c Casimir Pulaski University of Technology and Humanities, Radom, Poland

**Keywords:** Multiple sclerosis, Postural control, Posturography, Stability vector, Directional indices

## Abstract

**Background:**

Early and accurate diagnosis of multiple sclerosis (MS) is crucial for its effective treatment. In MS diagnostic, neuronal networks that control posture and movement are of particular importance, which performance can be assessed using static posturography. Unfortunately, most of the commercially available posturographic platforms are not equipped with the appropriate procedures.

**Methods:**

To solve this problem, the postural sway trajectories have been recorded in 55 MS patients while standing quiet with eyes open (EO), and then with eyes closed (EC). The trajectories were analyzed using our novel methods of postural sway parametrization, including sway stability vector (SV), anteroposterior and mediolateral sway indices (DIAP and DIML).

**Results:**

The results exhibited unique postural sway patterns that may be attributed to MS. Our novel parametrization methods of postural sway showed pathology specific increase of the postural sway velocity in EC tests. Additionally, we documented the abnormal alterations of the anteroposterior (AP) and the mediolateral (ML) sway indices that were also uniquely dependent on visual input. In EC tests, patients exhibited a characteristic pattern of sway increase in both AP and ML directions that correlated with the advance of the disease as measured by the EDSS Kurtzke scale and Functional System Scores.

**Conclusions:**

The applied in the present study our novel posturographic metrics give the assessment a diagnostic value. It allows us to recommend the static posturography test as a simple and safe supplementary clinical tool in the diagnosis of MS. In the assessment of MS pathology or the effects of its treatment, the impact of vision on the sway stability vector seems the most important factor.

## 1 Introduction

Multiple sclerosis (MS) continues to be a challenging and disabling pathology of the central nervous system but there is now a greater understanding of the underlying factors that drive the condition (Thompson et al., 2018). MS is an acquired disabling neurological disease of young adults. It affects approximately 2.3 million people worldwide (Doshi and Chataway, 2016). In MS, the brain immune system attacks the myelin sheath that covers axon fibers and causes nerve transmission problems. In consequence a heterogeneous array of symptoms and signs resulting from differential involvement of motor, sensory, visual, and autonomic systems (Doshi and Chataway, 2016). Escalation of the disease can cause permanent damage or deterioration of neuronal networks (Prosperini et al., 2014; Prosperini and Castelli, 2018). MS is frequently considered incurable, and available symptomatic treatments can only slow down its course. Emerging new therapies, however, can affect the course of the disease over time, by reducing the relapse rate, duration, and severity of attacks and its conversion into secondary-progressive MS (Brown et al., 2020). Additionally, physical therapy and exercise can help preserve remaining function and can help patients remain independent and mobile. From this perspective, simple but reliable monitoring of the MS course using the static posturography is for us a new challenge.

Early and accurate diagnosis of MS may be crucial for its successful treatment (Thompson et al., 2018). Magnetic resonance imaging is the main diagnostic method in MS diagnosis and therapeutic monitoring (Doshi and Chataway, 2016). Diagnosis can be additionally clarified by paraclinical data of evoked potentials (eg. visual or sensory) and results of the cerebrospinal fluid analysis (Doshi and Chataway, 2016; Deangelis and Miller 2014). Also, the static posturography revealed to be valuable in the MS clinic, particularly in the diagnosis of motor dysfunctions related to falls (Boes et al., 2012; Cameron and Lord, 2010; Inojosa et al., 2020a,b; Kalron et al., 2016a,b; Nilsagård et al., 2009; Pau et al., 2017; Prosperini et al., 2013; Prosperini and Castelli, 2018) Simplicity and safety of static posturographic tests make it attractive for use in the neurological clinic. Additionally, static posturography seems very sensitive to every change within the sensory-motor system which may be of key importance in the diagnosis and monitoring of MS (for review see Prosperini and Castelli, 2018). Results of the up to date studies relied, however, on traditional spatiotemporal sway indices that are not considered as reliable measures of postural stability, and should not be used for diagnostic purposes (Błaszczyk et al., 2003; Błaszczyk, 2008; Błaszczyk, 2016).

Static posturography is a simple non-invasive method used in contemporary labs and clinics to assess the quality of postural stability control (Błaszczyk, 2016). To maintain a stable erect posture, both in static and dynamic conditions the central nervous system must sense changes in our environment and base on this information must implement adequate patterns of muscular activation counteracting the loss of balance. This continuous process is controlled by partially redundant sensory inputs driven by proprioceptive, visual, and vestibular signals that closely interact with the motor output (Massion, 1992). Due to substantial nonlinearities, delays, and physiological limitations of the neuromuscular control the motor output of postural control is contaminated with noise in the form of random oscillations (Błaszczyk et al., 1993; Błaszczyk et al., 2020). The tremor-like spontaneous oscillations can be assessed during posturographic tests as changes in the pressure of feet on a support surface. The signal is known as the postural sway or the center of pressure (COP).

The COP sway signal is commonly used in contemporary labs and clinics to assess the physiological status of sensory and neuromuscular control in MS patients (Cameron and Lord 2010; Prosperini and Castelli, 2018). Due to the simplicity and safety of the force-plate posturography, there is increasing interest in using the method for neurological diagnostic, patients screening, pathology progressing, and fast assessment of treatment effects. So far, the diagnostic value of static posturography has been proved in the aging of the nervous system, and motor disorders and injuries. Recently, substantial research efforts focus on MS (Wajda et al., 2016; Prosperini et al., 2013; Inojosa et al., 2020a,b; Kalron et al., 2016a,b) Continuing this line of research we focused here on sway characteristics in MS. Multiple sclerosis signs and symptoms may differ greatly from patient to patient and throughout the disease depending on the location of affected nerve fibers. MS motor symptoms often include tremors, discoordination, and unsteady gait (Doshi and Chataway, 2016; Nilsagård et al., 2009; Pau et al., 2017; Thompson et al., 2018).

In this study, we aimed to test the applicability of our novel model of postural sway parametrization in the assessment of postural control decline in the course of multiple sclerosis. We hypothesized that the postural sway vector and directional sway indices can be sensitive to pathological damage to the neuromuscular networks that are symptomatic for MS. Towards this aim, we tested in a group of well diagnosed MS patients the impact of visual input on postural sway characteristics as measured by our novel metrics (Błaszczyk et al., 2014; Błaszczyk, 2016). The new metrics of postural sway are reliable measures of balance deficiencies that may impact stability in MS patients. In particular, they allow assessing the symmetry and muscle forces of lower extremities; they are also proved to be sensitive to muscle tremors and stiffness (Błaszczyk, 2016).

## 2 Material and methods

### 2.1 Experimental design

The study was approved by the Senate Ethics Committee of the Jerzy Kukuczka Academy of Physical Education. Research participants were patients of the University Clinical Center, the Silesian Medical University in Katowice. Fifty-five subjects (41 females and 14 males, age range: 20–53 years), volunteered to participate in the experiment. The only inclusion criterion was the ability to stand independently. All participants met the ethical and health conditions requirements for the posturographic study; they were capable to maintain erect posture for 60 s both with and without visual control. The patients provided their written informed consent to participate in this study. Their overall disability was assessed in the Expanded Disability Status Scale (EDSS) and the type and severity of neurologic impairment were evaluated by Kurtzke Functional System Score (FSS) (Kurtzke, 1983; Hobart et al., 2000; Kalron et al., 2016a,b). For analysis purposes, the patients' data were grouped according to their EDSS scale. Results of static posturography tests from age- and sex-matched group of the healthy subjects were used as reference data (Błaszczyk and Cieślińska-Świder, 2019; Cieślińska-Świder and Błaszczyk, 2019). The data are gathered in Table 1.

**Table 1:** Characteristics of the experimental groups.

Group	EDSS	N	Mean age (yrs)
G1	1–2	M = 6, F = 16	33.5 ± 9.9
G2	2.5–3.0	M = 4, F = 12	32.9 ± 9.6
G3	3.5–4.5	M = 4, F = 13	42.5 ± 8.6

EDSS - Expanded Disability Status Scale, N – number of M - male, and F - female patients.

During the experimental session, each patient performed six 25.6 s trials separated by short breaks to avoid fatigue or boredom. The trials were performed alternately with and without visual feedback. Patients'postural sway in the form of the center of foot pressure (COP) trajectories during quiet stance had been recorded with eyes open (EO) and eyes closed (EC). Each trial was repeated 3 times. During testing, subjects were standing barefoot on the force plate (QFP Medicapteurs, France) with their heels aligned and arms kept comfortably at the side.

### 2.2 Novel methods of COP signals analysis

The COP trajectory signals were filtered at 10 Hz and sampled with a 16-bit A-to-D interface with a frequency of 40 Hz. The trajectory was characterized with main sway parameters including anteroposterior (V_AP_), mediolateral (V_ML_), and total (Vamp) COP velocities (Raymakers et al., 2005; Błaszczyk, 2016). Based on these measures sway directional indices (DIAP and DIML), and sway stability vector (SV) were computed using Microsoft Office Excell 2007 with custom scripts. To characterized balance control symmetry, the sway directional indices and sway stability vector were computed according to the following formulas (Błaszczyk et al., 2014, 2016; Błaszczyk, 2016):

(1) DIAP = V_AP_/V_amp_

(2) DIML = V_ML_/V_amp_

The COP sway velocities were also used to compute the sway vector (SV) coordinates (Błaszczyk, 2016). The amplitude of the vector (SVamp) was equal to the mean COP velocity:

(3) SVamp = Vamp

whereas its azimuth (SVaz) was defined as:

(4) SVaz = arctan(V_AP_/V_ML_)

All statistical analyses were performed using Statistica v. 6.0 software (Statsoft U.S.A). Repeated measures analysis of variance (ANOVA) was used to determine statistically significant differences between the patients’ group and the two visual conditions (EO vs. EC). Spearman Rank Order Correlations between the COP sway measures and the EDSS and FS scales were assessed across conditions. A *P*-value of smaller than 0.05 was considered significant.

## 3 Results

### 3.1 Sway vector (amplitude SVamp, and azimuth SVaz)

**Figure 1:**
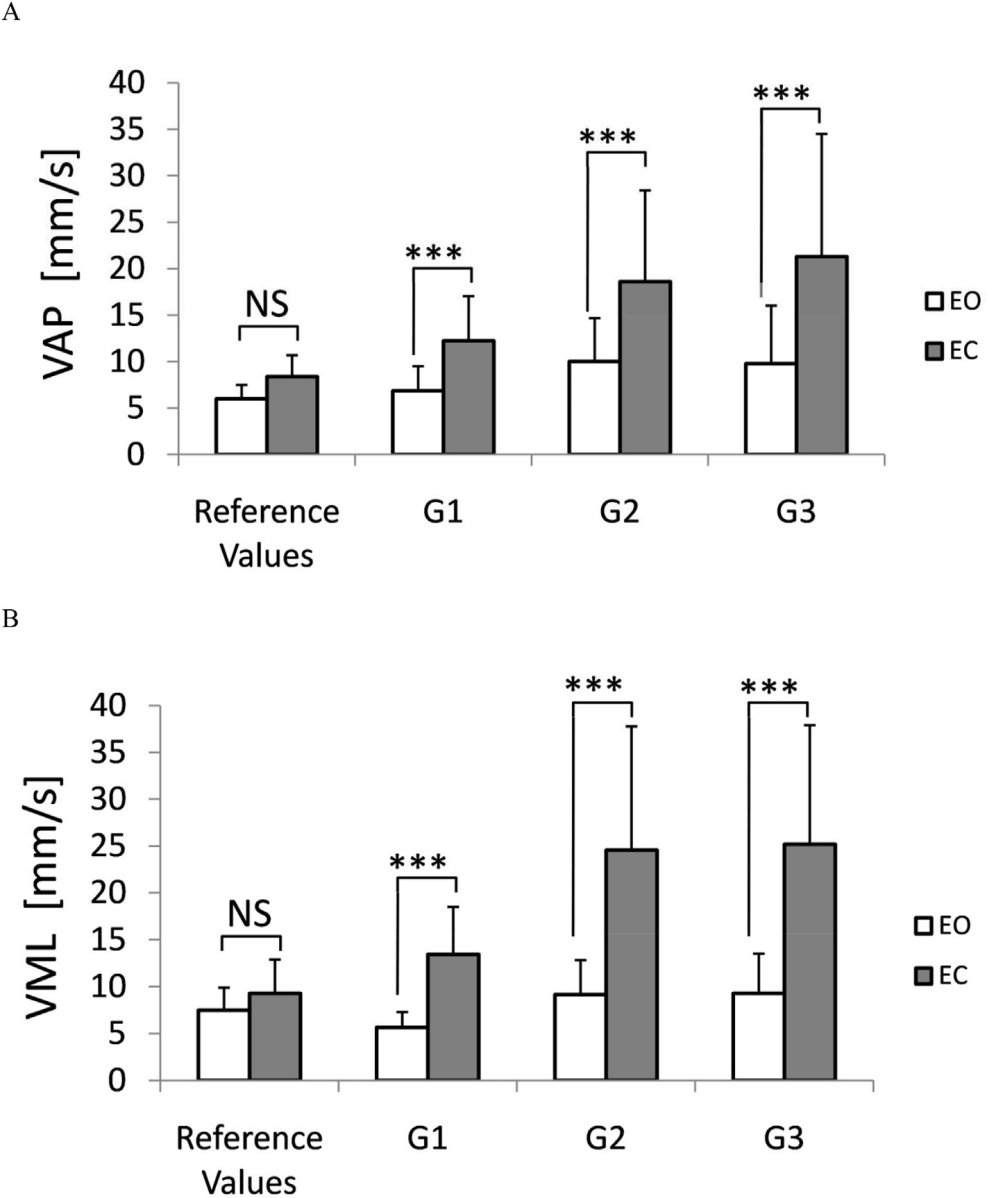
Changes of mean anteroposterior AP COP velocity (panel A) and mediolateral ML COP velocity (panel B) in 3 groups of MS patients (G1-G3; for details see Table 1). Tests were performed with eyes opened (EO), and with eyes closed (EC). Reference values were computed from the age-matched, healthy control group (n = 36). Error bars represent standard deviation. NS – insignificant difference, ∗∗∗-p≤0.001.

The 3 × 2 ANOVA with the 'group' and 'vision' as grouping factors showed the main effect of both the group (F_2,52_ = 6.56; *P* ≤ 0.005) and the vision (F_1,52_ = 93.87; *P* ≤ 0.001) for the VCOP that represents sway vector amplitude (SVamp). The interaction of both factors also reached the level of significance (F_2,52_ = 4.45; *P* ≤ 0.02). The post-doc test showed a highly significant increase of SVamp values in all MS groups in EC trials to compare with the test with full sensory control (EO trials). Importantly the SVamp in EO tests in the group G2 and G3 remained at the level typical for healthy subjects (15.1 ± 7.4 mm/s vs 14.9 ± 2.8 mm/s for patients and healthy population, respectively). Postural control in the G1 group was characterized by a much lower SVamp (9.9 ± 3.1 mm/s). Eye closure resulted in an extraordinary increase of the mean increase of SVamp ranging 85% for all groups, whereas the observed increase was correlated with the advance of the disease ranging 70.6% in the G1 group, 82.4% in G2, and 100.4% in the group G3. Such an effect has not been observed in the reference group of young adults, where the increase due to the elimination of visual feedback was insignificant. The results of this analysis are shown in Figure 1A.

**Figure 2:**
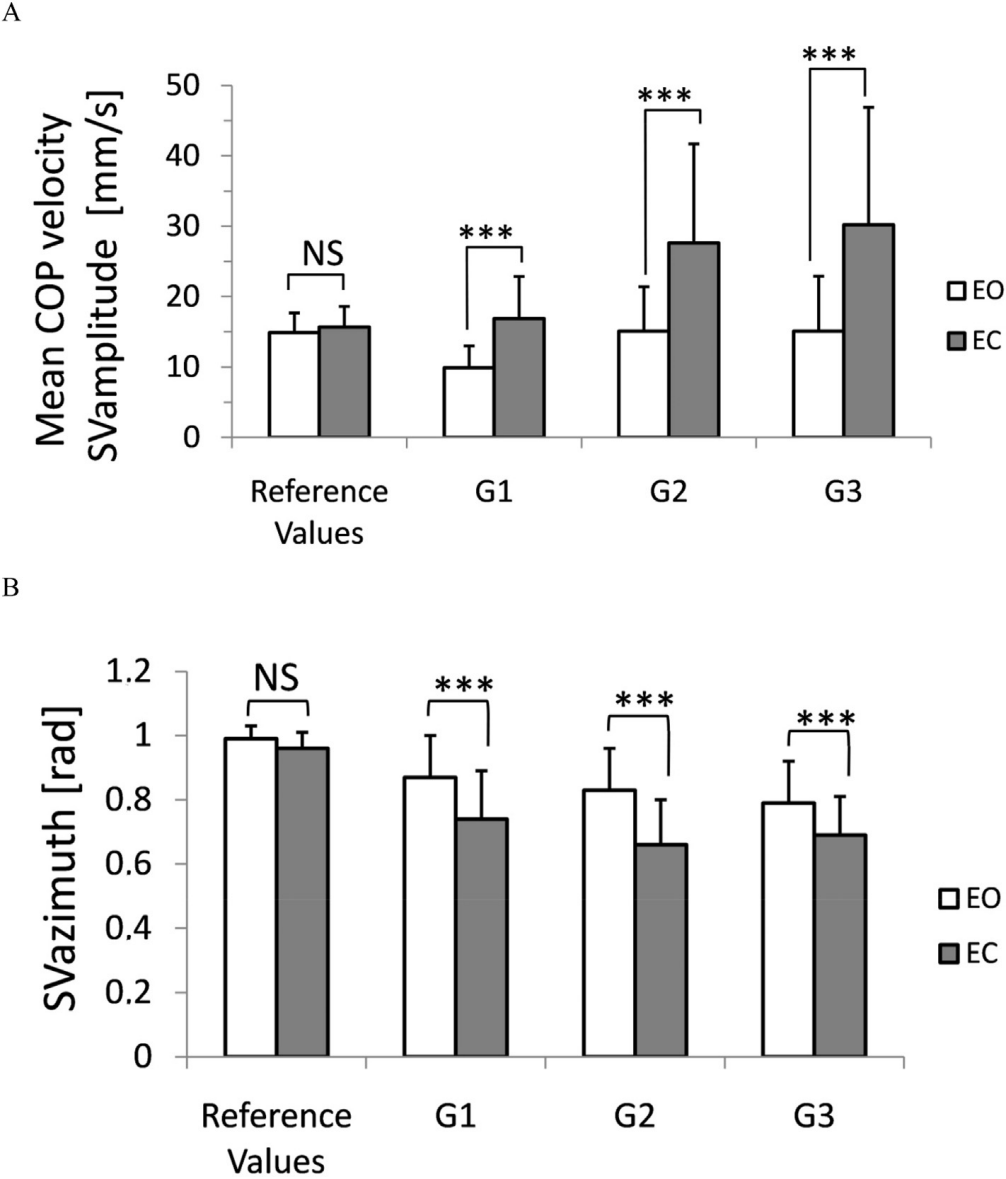
Attributes of Stability Vector: amplitude - SVamp (panel A), and azimuth – Svaz (panel B) in 3 groups of MS patients (G1-G3; for details see Table 1). Posturographic tests were performed with eyes opened (EO), and with eyes closed (EC). Reference values were computed from the age-matched, healthy control group (n = 36). Error bars represent standard deviation. NS – insignificant difference, ∗∗∗-p≤0.001.

The next analysis concerned sway vector azimuth (SVaz). Group-by-vision ANOVA showed only a significant effect of vision factor only (F_2,52_ = 111.4; *P* ≤ 0.0001). The posture of MS subjects while tested without visual input was characterized by a significant decrease of the SVaz to compare with posture controlled with full sensory inputs (EO trials in Figure 2). The observed mean drop was similar in all MS groups and ranged 16%. These results differed from the healthy young adults' group (the reference values in Figure 1) where the drop of the SVaz due to vision exclusion remained at the level of 3%. However, both values of SVaz in EO and EC trials observed in the patients’ groups were significantly lower to compare with the reference values. For details see Figure 1B.

### 3.2 Anteroposterior (V_AP_) and mediolateral (V_ML_) postural sway velocity

The ANOVA with the 'group' and 'vision' as grouping factors showed the main effect of both the group (F_2,52_ = 4.47; *P* ≤ 0.02) and the vision (F_1,52_ = 84.58; *P* ≤ 0.0001) for the V_AP_ velocity. The interaction of both factors also reached the level of significance (F_2,52_ = 3.94; *P* ≤ 0.03). The most symptomatic for all experimental groups was a highly significant increase in V_AP_ while tested with EC (*P* ≤ 0.0005). In the population of healthy young adults maintaining the standing posture without visual feedback (EC test) results in the usually insignificant, increase of the V_AP_. In contrast to the reference control group the observed increase of the V_AP_ while tested with EC ranged from 79% in G1, 86% in G2 group, and up to 118% in the G3 group. For details see Figure 2A.

A more significant increase of balance asymmetry was observed in the V_ML_ in all MS patients groups. Analysis of variance with showed the main effect of both the group (F_2,52_ = 6.56; *P* ≤ 0.005) and the vision (F_1,52_ = 93.87; *P* ≤ 0.001) for the V_ML_. The interaction of both factors was also significant (F_2,52_ = 4.45; P ≤ 0.02). The observed increase of the V_ML_ observed in response to eyes closure range almost 138% in G1 (an increase from 5.6 – 13.4 mm/s), 169% (from 9.1 – 24.6 mm/s) in G2. A similar increase (172%) within a similar range was observed in the G3 group (9.3 in EO trial and 25.2 in EC). The results are depicted in Figure 2B.

### 3.3 Sway directional indices (DIAP and DIML)

**Figure 3:**
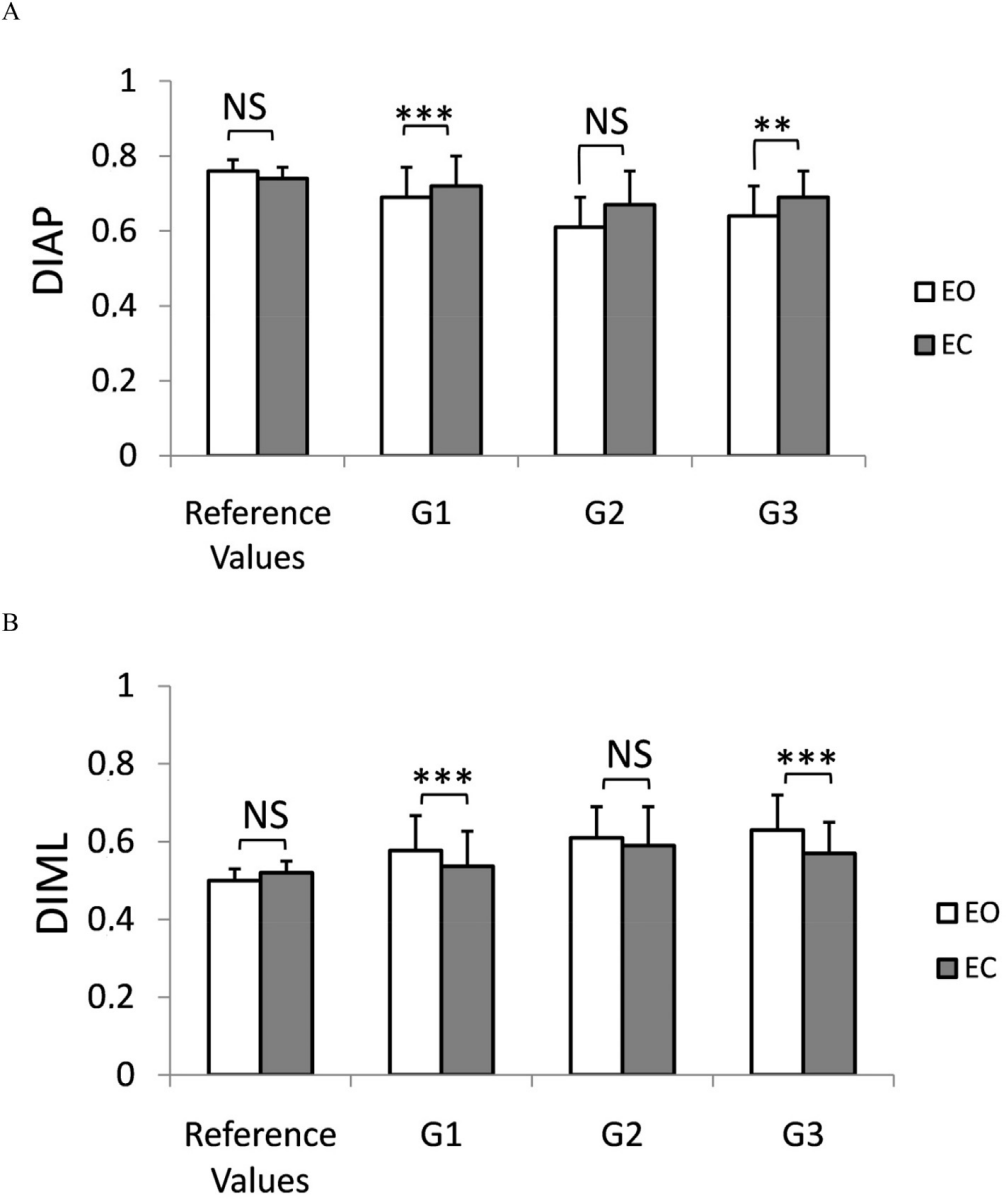
Changes of Directional Sway Indices: DIAP panel A, and DIML panel B) in 3 groups of MS patients (G1-G3; for details see Table 1). Standard posturographic tests were performed with eyes opened (EO), and with eyes closed (EC). Reference values were computed from the age-matched, healthy control group (n = 36). Error bars represent standard deviation. NS – insignificant difference, ∗∗- p ≤ 0.01 ∗∗∗-p≤0.001.

The ANOVA confirmed a significant effect of ‘vision’ (F_1,52_ = 16.44; P ≤ 0.001) for the DIAP. The posthoc test showed significantly higher DIAP values in G1 and G3 groups in EC tests. In the G1 group the DIAP increase from 0.69 ± 0.08 (EO) to 0.72 ± 0.08 (EC), P ≤ 0.01. Similarly, eye closure resulted in an increase in the DIAP value in the G3 (from 0.64 ± 0.08 up to 0.69 ± 0.08 for EO and EC trials, respectively. In contrast, such a significant increase in the DIAP was not observed in G2 patients. Changes are depicted in Figure 3A.

For the DIML, a main effect of vision (F_1,52_ = 19.33; *P* ≤ 0.0001) was also documented. MS patients from G1 and G2 groups exhibited lower values in EO trials to compare with a quiet stance with EC. Eyes closure resulted in a decrease of the mediolateral directional index but the effect was insignificant in patients of the G2 group (EDSS 1.5–2.5). In the G1 group the DIML decrease from 0.58 ± 0.09 (EO) to 0.54 ± 0.1 (EC), *P* ≤ 0.005. Testing with EC resulted also in a decrease of DIML value in the G3 from 0.63 ± 0.09 (EO) to 0.57 ± 0.1 (EC); *P* ≤ 0.005. For details see Figure 3B.

### 3.4 Correlations analysis

Spearman Rank Order Correlations test revealed moderate correlations between EDSS, Kurtzke Functional System Scores, and almost all sway measures including V_AP_, V_ML_, Vamp, and Directional Sway Indices (DIAP and DIML). The most significant correlations were found between Kurtzke Functional System Score (FSS) and the aforementioned sway measures. Particularly correlations between pyramidal (FSP) and cerebellar (FSC) functional systems were most evident. In particular, the FSP and FSC correlated significantly with ML velocity while the postural test was performed with “eyes closed” (0.58 and 0.45; *P* ≤ 0.0005, respectively). Also, Sway Vector Amplitude (SVamp) both in EO and EC tests correlated positively with both FSs (*P* ≤ 0.0005). The results of correlation analyses are gathered in Table 2.

**Table 2:** Spearman Rank Order Correlations (R and *P*-level) between main posturographic measures and Expanded Disability Status Scale (EDSS) and Kurtzke Functional System Score (FSS) in 55 Multiple Sclerosis patients.

R	EDSS	Kurtzke Functional System Score (FSS)			
p≤		Visual	Brainstem	Piramidal	Cerebellar
V_ML_ EO	**0.41**	**-0.31**	**0.37**	**0.47**	**0.49**
	0.002	0.02	0.01	0.0003	0.0002
V_ML_ EC	**0.39**	**-**	**0.34**	**0.58**	**0.45**
	0.003	-	0.01	0.00001	0.0005
V_AP_ EO	**-**	**-0.3**	**0.31**	**0.33**	**0.32**
	-	0.03	0.02	0.01	0.02
V_AP_ EC	**0.29**	**-0.28**	**0.29**	**0.48**	**0.37**
	0.04	0.04	0.04	0.0002	0.005
SVamp EO	**0.32**	**-0.32**	**0.34**	**0.4**	**0.41**
	0.02	0.02	0.01	0.003	0.002
SVamp EC	**0.34**	**-0.30**	**0.32**	**0.55**	**0.43**
	0.02	0.03	0.02	0.00001	0.001
SVaz EO	**-0.32**	**-**	**-**	**-0.38**	**-0.30**
	0.02	-	-	0.005	0.03
DIAP EO	**-0.32**	**-**	**-**	**-0.38**	**-0.29**
	0.02	-	-	0.005	0.03
DIML EO	**0.33**	**-**	**-**	**0.38**	**0.29**
	0.02	-	-	0.005	0.03

Significance of correlation is provided as p-value

## 4 Discussion

The present study aimed to verify the applicability of our new model of the postural sway parametrization in the assessment of neuromuscular control impairments due to multiple sclerosis. There are two of the most important discoveries of the research presented here. Firstly, Using new sway metrics we can characterize the unique pattern of changes in postural control, and secondly, the observed changes in sway were consistent with the progression of the disease.

Postural control base upon the interaction of many neuronal systems that are driven by the continuous inflow of sensory information (Massion, 1992). Visual and vestibular systems are two inputs of the top-down control. The third main input is the proprioception from the joints and muscles of the lower extremities. In particular, ankle joint proprioception plays a fundamental role in maintaining erect posture (Błaszczyk et al., 1994; Błaszczyk et al., 2020). Sensorimotor signals are transmitted here by the longest and thickest myelinated nerve fibers which activity requires a lot of energy. Consequently, they are prone to aging, damage, or energy deficit (Błaszczyk, 2018). Due to this distributed control, posture and its physiological measures are sensitive to various physiological and pathological changes in MS that can be assessed in static posturography (Cameron and Lord, 2010; Inojosa et al., 2020a,b; Kalron et al., 2016a,b; Prosperini and Castelli, 2018).

The stability of postural balance control can be assessed based on several metrics. These basic output parameters of static posturography include an envelope of the postural sway trajectories and sway velocity. These metrics describe primarily the biomechanical asymmetries in balance control. The first one has several drawbacks that have been eliminated in our recently introduced models: the sway directional indices and the sway stability vector (Błaszczyk et al., 2014; Błaszczyk, 2016). In contrast to standard measures that allow assessing natural asymmetries of postural control base on normalized values that are generally insensitive to the length of a trial. They provide reliable measures of postural control based on a single 30-second trial (Błaszczyk et al., 2016).

Until recently, the main limitation of the static posturography as a medical diagnostic method in neurodegenerative diseases was the lack of reliable standards for the postural sway assessment that can be used by clinicians. For many years only the spatiotemporal sway parameters were used in MS to assess the postural control (Prosperini et al., 2014; Boes et al., 2012; Inojosa et al., 2020a,b). Particularly, the mean COP velocity only seemed to be such a gold standard (Raymakers et al., 2005; Błaszczyk, 2016). Recently several standardized and reliable measures have been introduced (Błaszczyk and Orawiec, 2011; Błaszczyk et al., 2014, 2016; Błaszczyk, 2016). Particularly the sway vector (SV) and sway directional indices (DIAP and DIML) have been proved to be veridical measures of neuromuscular control of the erect posture (Błaszczyk et al., 2014; Błaszczyk, 2016). In particular, they allowed documenting the age-related and pathology-related changes in postural sway in older adults and patients with Parkinson's disease that were characterized by an increase of the mediolateral sway (Błaszczyk et al., 2007; Błaszczyk and Orawiec, 2011). In contrast to the aforementioned populations, the MS patients exhibit different and pathology specific changes in postural sway (for review see Prosperini and Castelli, 2018) which are characterized by substantially higher COP velocities in EC tests that correlated with the progression of the disease what is consistent with previous findings (Inojosa et al., 2020a,b; Prosperini et al., 2014). The exclusion of visual input differently affected DIAP and DIML. These unique for MS changes could be also observed in Sway Vector azimuth (SVaz). This pattern of changes is different from normal, age-matched, healthy adults (Błaszczyk and Cieślińska-Świder, 2019; Cieślińska-Świder and Błaszczyk, 2019).

The changes of the postural sway observed in our study can be explained by the demyelination of neuronal networks characteristic of the MS (Lucchinetti et al., 2000; Arancibia-Carcamo and Attwell, 2014; Kalron et al., 2016; Prosperini et al., 2014; Maranzano et al., 2019; Monaco et al., 2020). Four fundamentally different processes, such as autoimmunity or virus infection, may induce MS-like inflammatory demyelinating plaques and suggest that MS may be a disease with heterogeneous pathogenetic mechanisms (Lucchinetti et al., 2000). Recently has been recognized, however, that in long axonal fibers of the motor network, the process of demyelination is usually initiated by the energy-deficient unsealing of the axonal-glia junctions (Howell et al., 2010; Perry and Teeling 2013). Particularly, a deficit of the functional-metabolic interaction may initiate the axonal degeneration leading to apoptosis in the involved neurons (Błaszczyk, 2020). Apoptosis occurs according to a strictly ordered set of biochemical events, one of which is a characteristic reduction in neuron and axonal volume (Pasantes-Morales and Tuz, 2006). Local deficits in energy metabolism may result in axonal volume changes. The axonal shrinkage results in the unsealing of neuro-glial junctions at the Ranvier's nodes. As a result, potassium ions outflow from paranodal niches attracts microglia which activity escalates the demyelination process (Howell et al., 2010; Perry and Teeling, 2013). Consequently, apoptosis may contribute to neuronal loss and impairment of the posture-motor network. The pathological process strikes firstly neurons of the postural and sensorimotor systems and therefore the changes can be observed in the postural sway. The pathogenetic heterogeneity of plaques and particularly the contribution of energy metabolism in the process of demyelination may have fundamental implications for the MS diagnosis, therapy, and rehabilitation of the disease.

The impairment of the neuronal activity results in a decline in energy metabolism in the axonal fibers and thus escalate the damage. Focal lesions of myelin on individual fibers usually cause an inflammatory reaction and are not easily repaired unless we restore axonal electrical conductivity (Howell et al., 2010). The damaged axon and its synapses are reduced by a mechanism dependent on the activity of the SARM1 protein, and mitogen-activated kinases (MAPK) (Essuman et al., 2017; Gerdts et al., 2016; Walker et al., 2017). Interestingly, the nicotinamide-nucleotide adenylyltransferase (NMNAT2) enzyme in the NAD recovery pathway can restore damaged, energetic metabolism, and thus reverse the synaptic-axonal degeneration (Walker et al., 2017). All pathological changes result in increased delays within postural/motor control and finally augments the postural sway. The changes, however, are well compensated by the vision in the early stage of MS (Inojosa et al., 2020a,b). As documented here the pathological increase of swaying velocity was observed in EC trials, only. This makes the standing posture of MS patients less stable and prone to falls (Cameron and Lord, 2010; Nilsagård et al., 2009; Prosperini et al., 2013).

Most MS patients experience muscle weakness in their extremities and difficulty with coordination and balance. The neuronal network that controls movement, balance, and postural stability seems very sensitive to changes in transmission speed which determines the amount of postural sway. Current research has shown that pathological changes in multiple sclerosis have a significant impact on the directional parameters DIAP and DIML and on the azimuth of the sway stability vector. In particular, these results document changes in the asymmetry of postural control, the consequence of which is the increased susceptibility to the postural balance deficit (Błaszczyk et al., 2016) that is symptomatic in most patients with MS. DIAP changes are also characteristic for altered neuromuscular control, and in particular for reduced strength and increased stiffness of the muscles stabilizing the ankle joint. As long as the amount of sway remains below the critical value it does not threaten balance and does not restrain the movement control (Błaszczyk et al., 1993). Redundancy and interaction of proprioceptive, visual, vestibular systems ensure erect posture stability while the postural sway does not interfere with the control. In young healthy adults, even turning off the visual input does not interfere with the postural control (Błaszczyk and Cieślińska-Świder, 2019; Cieślińska-Świder and Błaszczyk, 2019). Eyes closure results in a limited and statistically insignificant increase of postural sway. In contrast, such a destructive impact of the visual input exclusion is pronounced in MS patients (Inojosa et al., 2020a,b). Depending on lesion size and its location the sway increase may exceed 100% of the EO values. Importantly in habitual stance with full sensory control (EO test), patients' postural sway was not substantially different from normal. While tested without visual input (EC test) sway characteristics of the MS patients exhibited a strong dependence on pathology, its localization, and the size of the lesion (Boisgontier et al., 2017). The magnitude of changes correlated with the EDSS scale and Kurtzke Functional System Score, which underline the diagnostic value of static posturography (Inojosa et al., 2020a,b). Interestingly, our results are consistent with previous findings (Kalron et al., 2016a,b) and confirm that only the Functional Systems involved in the postural/motor control exhibited such correlations. Thus, the static posturography applied in MS may help to identify ongoing subclinical progression and provide a valuable measure to monitor response to therapies. In conclusion, the results of the present study documented a unique, pathology-dependent pattern in postural sway changes. It seems that all these changes can be easily monitored with posturographic testing along with MRI. This allows us to recommend static posturography complemented with our new methods of postural sway parametrization as a valuable clinical supplementary tool in the diagnosis and monitoring progress of multiple sclerosis.

## Declarations

### Author contribution statement

Janusz W. Błaszczyk: Conceived and designed the experiments; Wrote the paper.

Joanna Cieślińska-Świder: Performed the experiments; Analyzed and interpreted the data.

Renata Orawiec: Performed the experiments; Contributed reagents, materials, analysis tools or data.

### Funding statement

This work was supported by the Jerzy Kukuczka Academy of Physical Education in Katowice.

### Data availability statement

Data will be made available on request.

### Declaration of interests statement

The authors declare no conflict of interest.

### Additional information

No additional information is available for this paper.
